# Antigen-specific T cell redirectors (ATR) for antigen-specific redirection of T cells to tumors

**DOI:** 10.1186/2051-1426-2-S3-P36

**Published:** 2014-11-06

**Authors:** Christian Schütz, Karlo Perica, Juan Varela, Carl Haupt, Mathias Oelke, Jonathan Schneck

**Affiliations:** 1 Johns Hopkins School of Medicine, Department of Pathology, Institute for Cell Engineering, United States; 2 Johns Hopkins School of Medicine, Department of Biomedical Engineering, Institute for Cell Engineering, United States; 3 Johns Hopkins School of Medicine, United States

## 

Immunotherapy is the modulation of a patient's immune system to treat illness. Unfortunately many T cell based attempts have failed due to the fact that existing tumor-specific T cells are mostly anergic or tolerized and ex vivo generated T cells are often already of exhausted phenotype. Therefore, investigators have developed alternative approaches including bispecific antibody technology to redirect fully functional non-tumor specific T cells to the tumor. This has been primarily accomplished through targeting CD3, which is expressed on all T cells to engage and redirect them towards a molecule that is expressed on the tumor cells. Here we present a novel nanoparticle based approach to selectively target cytotoxic T cells (CTL) and re-direct them to kill tumors, termed ATR (Antigen-specific T cell Redirectors).

ATR were generated by coupling either MHC-Ig dimer or clonotypic anti-TCR antibody 1B2 to target the effector T cell population and an anti-CD19 to re-direct those to CD19+ tumor target cells onto 50-100nm nanoparticles. Flow cytometry and microscope based data confirm that the described ATR phenotype efficiently and stably stain tumor and T cells in a dose dependent manner, and ATR mediate antigen-specific conjugate formation of effector T cells and tumor target cells. We further developed two clinically relevant protocols to test and optimize our ATR in vitro. First a pre-treatment approach in which the effector T cells are pre-incubated with ATR mimicking an adoptive transfer approach and second a co-culture protocol that mimics an active immunotherapy approach of direct ATR injection. Antigen-specific ATR mediated re-direction of T cells to tumor target cells was demonstrated in ^51^Cr-release killing assays at low E:T ratios. Variation of ATR target-cell: effector-cell targeting molecule ratio could further increase efficacy. Finally, intra tumoral ATR injection induced T cell re-direction and reduced tumor growth in a s.c. Raji/SCIDbeige treatment model.

In summary this data demonstrates that ATR target and redirect antigen-specific CTL to tumor cells that would otherwise not be recognized and mediates their lysis. ATR can be used to develop new innovative immunotherapeutic approaches for all cancers that can be targeted with antibodies or antibody-like molecules. Furthermore, ATR could also be used in conjunction with virus-specific immunization to specifically increase the targeted CTL population. Ultimately, we expect ATR and their potential for clinical applications to increase our understanding of tumor immunotherapy through T cell redirection.

